# Salicylic Acid Treatment Alleviates the Heat Stress Response by Reducing the Intracellular ROS Level and Increasing the Cytosolic Trehalose Content in Pleurotus ostreatus

**DOI:** 10.1128/spectrum.03113-22

**Published:** 2022-12-12

**Authors:** Guang Zhang, Peng Yan, Doudou Leng, Li Shang, Chaohui Zhang, Zhongwei Wu, Zhenhe Wang

**Affiliations:** a College of Life Science and Technology, Henan Institute of Science and Technology, Henan, Xinxiang, People′s Republic of China; South China Agricultural University

**Keywords:** heat stress, Ca^2+^, ROS, salicylic acid, trehalose

## Abstract

Pleurotus ostreatus is usually cultivated in horticultural facilities that lack environmental control systems and often suffer heat stress (HS). Salicylic acid (SA) is recognized as a plant defense-related hormone. Here, SA treatment (200 μM) induced fungal resistance to HS of *P. ostreatus*, with decreased malondialdehyde (MDA) content and *HSP* expression. Further analysis showed that SA treatment in *P. ostreatus* increased the cytosolic trehalose content and reduced the intracellular reactive oxygen species (ROS) level. Moreover, H_2_O_2_ could restore the MDA content and *HSP* expression of *P. ostreatus* treated with SA under HS. In addition, trehalose (25 mM) or CaCl_2_ (5 mM) treatment induced fungal resistance to HS, and CaCl_2_ treatment increased the cytosolic trehalose content of *P. ostreatus* under HS. However, inhibiting Ca^2+^ levels using Ca^2+^ inhibitors or mutants reversed the trehalose content induced by SA in *P. ostreatus* under HS. In addition, inhibiting trehalose biosynthesis using *Tps*-silenced strains reversed the MDA content and *HSP* expression of *P. ostreatus* treated with SA under HS. Taken together, these results indicate that SA treatment alleviates the HS response of *P. ostreatus* by reducing the intracellular ROS level and increasing the cytosolic trehalose content.

**IMPORTANCE** Heat stress (HS) is a crucial environmental challenge for edible fungi. Salicylic acid (SA), a plant defense-related hormone, plays key roles in plant responses to biotic and abiotic stresses. In this study, we found that SA treatment increased the cytosolic trehalose content and induced fungal resistance to HS in *P. ostreatus*. Further analysis showed that SA can alleviate the HS of *P. ostreatus* by reducing the intracellular ROS level and increasing the cytosolic trehalose content. Our results help to understand the mechanism underlying the responses of *P. ostreatus* to HS. In addition, this research provides new insights for the cultivation of *P. ostreatus*.

## INTRODUCTION

Pleurotus ostreatus (Jacq.:Fr.) Kumm, known as oyster mushroom, is a food ingredient famous for its nutritional value and unique flavor. Rich in nutrients, *P. ostreatus* possesses medicinal and functional value in the human diet due to its antitumor (1), antioxidant (2), and antifungal properties (3) and its ability to reduce hypercholesterolemia (4). In recent years, *P. ostreatus* has become a promising nutraceutical candidate due to its therapeutic benefits on hypertensive patients (5). Studies have revealed that polysaccharides are the main bioactive components among the various compounds extracted from *P. ostreatus* (6) and are responsible for its physiological responses.

Due to its commercial and medicinal value, *P. ostreatus* is becoming one of the most common artificially cultured mushrooms worldwide. According to the statistics of the Chinese Edible Fungi Association, the yield of *P. ostreatus* was the third largest among commercially cultivated edible fungi in China (7). However, unlike the industrial cultivation of *Agaricus bisporus*, *P. ostreatus* is mainly cultivated using the traditional agricultural cultivation mode in developing countries. Its cultivation usually adopts the traditional bag cultivation method in horticultural facilities that lack environmental control systems, such as plastic sheds or greenhouses (8). Fungal growth undergoes external environmental changes, causing a decline in the quality of *P. ostreatus*. During fungal growth and developmental processes, *P. ostreatus* often suffers from thermal stress, such as heat stress (HS), in summer that seriously affects its production and quality (9). Understanding the thermal responses of *P. ostreatus* is necessary and provides critical guidance for its cultivation.

HS is one of the abiotic stresses that affect the cultivation of edible fungi (10). Accumulated reactive oxygen species (ROS) (10), apoptotic-like cell death (11), impaired cell wall integrity and structure (8), spawn burning (12) and *Trichoderma* contamination (13) have been reported in *P. ostreatus* that undergoes HS. The heat stress response (HSR) of mushrooms is becoming a hot research topic and has attracted widespread attention recently. The physiological and metabolic changes of *P. ostreatus* exposed to HS are extremely complex and affect fungal growth and development as well as the interactions between cells and molecules (9). Extracellular metabolites (14) and differentially expressed proteins (DEPs), including heat shock proteins (HSPs) (15), have been identified in the HSR of *P. ostreatus*. Subsequently, a series of genes and metabolic products were found to play important roles in the HSR of *P. ostreatus*, such as alternative oxidase (9) and zinc cluster proteins (7). In addition, the nonreducing disaccharide trehalose induced by ROS signaling can alleviate HS by inhibiting glycolysis and stimulating pentose phosphate pathway activity in *P. ostreatus* (16). Moreover, signaling molecules, including ROS species (10) and nitric oxide (17), are believed to play moderating roles in the HSR of *P. ostreatus*. These studies reveal that extracellular metabolites, DEPs, metabolic products, and signaling molecules are critical for the adaptation of *P. ostreatus* exposed to HS. However, the mechanism of HSR in *P. ostreatus* remains unclear.

The phenolic compound salicylic acid (SA), a beta hydroxyl phenolic acid and signaling molecule, is known as a plant defense-related hormone that is widely produced in prokaryotes and plants (18). Intensive research has demonstrated that SA plays crucial roles in plant responses to biotic and abiotic stresses, such as pathogenic attacks (18) and thermal stress (19). In addition, SA functions well in the homeostasis, metabolism, and growth of plants (18). Unfortunately, the functions of SA are not well studied in fungi, especially in basidiomycetes. In *P. ostreatus*, acetylsalicylic acid in the extracellular fluid shows an increase in concentration after exposure to high temperature (14). Furthermore, it is noteworthy that SA changed 29-fold, with the second largest change multiple among all metabolites of *P. ostreatus* during HS (20). These studies indicate that SA might play a crucial role in the HSR of *P. ostreatus*.

In the present study, we explored the function of SA treatment in the HSR of *P. ostreatus*. Our results showed that SA treatment regulated mycelial growth and trehalose biosynthesis in *P. ostreatus*. Further analysis indicated that SA treatment could alleviate HSR in *P. ostreatus* by reducing the intracellular ROS level and increasing the cytosolic trehalose content.

## RESULTS

### SA treatment promoted fungal growth and induced fungal resistance to HS.

*P. ostreatus* was cultured on complete yeast medium (CYM) plates supplemented with SA to investigate the effect of SA on mycelial growth. Low concentrations of SA treatment (0–500 μM) promoted fungal growth (Fig. S1A in the supplemental material). SA treatment at 200 μM increased the mycelial diameter and biomass of the wild-type (WT) strain by 19.10% and 42.24%, respectively, compared with that of the zero control (Fig. S1B and C). A precise examination showed that the fungal hyphae of the WT strain treated with SA were highly branched, and the distance between two branches was decreased by 54.37% compared with that of the WT strain (Fig. S2B).

**FIG 1:**
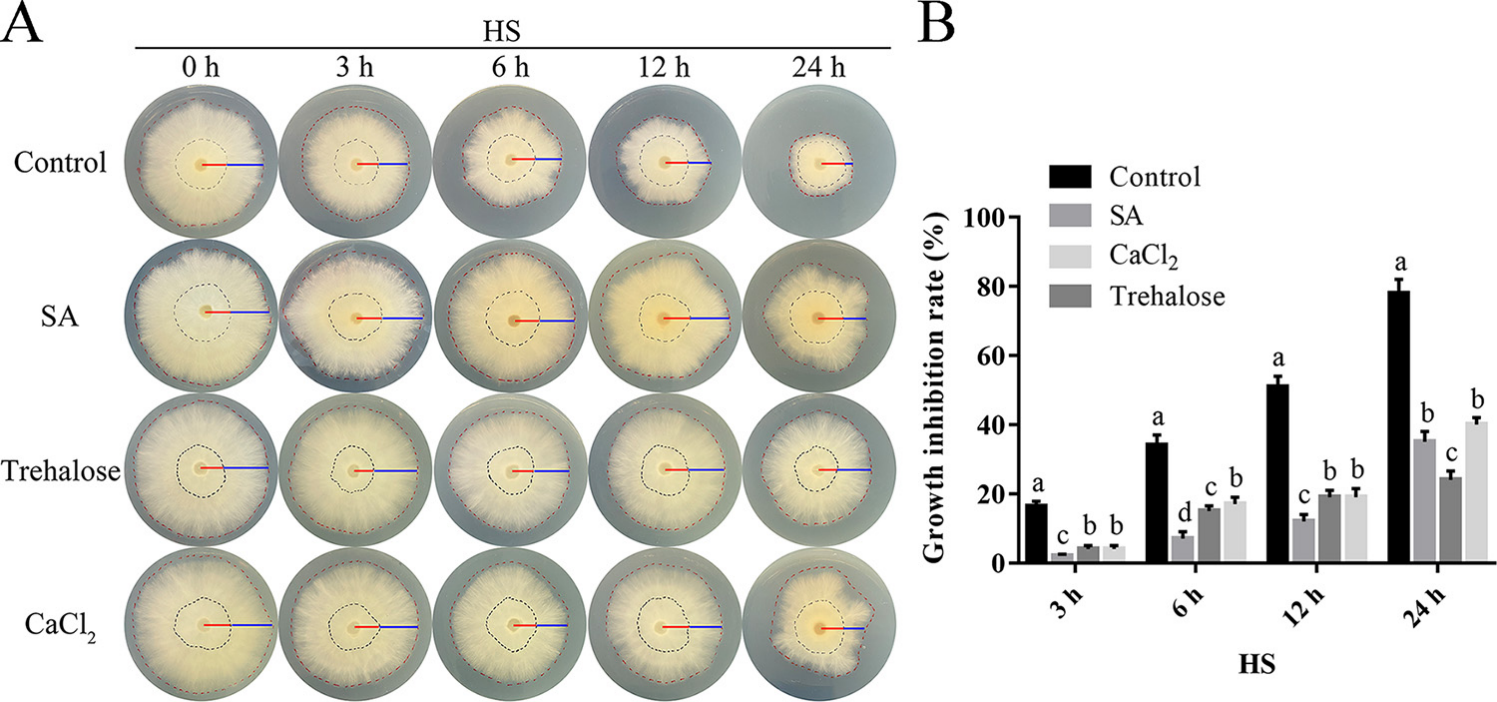
SA treatment induced fungal resistance against HS in *P. ostreatus*. The WT strain was cultured on CYM plates supplemented with SA (200 μM) at 28°C for 3 days and then treated at 40°C for 3, 6, 12, or 24 h, after which the fungi were cultured at 28°C to recover for 3 days. Trehalose (25 mM) and CaCl_2_ (5 mM) were added just before HS treatment. ddH_2_O containing SA, trehalose, and CaCl_2_ was used to formulate CYM medium, and pure ddH_2_O was used as a control. (A) Fungal growth of *P. ostreatus*. The red lines indicate the mycelial radius before HS, and the blue lines indicate the mycelial radius after HS. (B) Fungal growth inhibition rates. Three independent biological replicates were performed for all experiments. The values are interpreted as the mean ± SD. The standard deviations are indicated by error bars. The small letters indicate significant differences between the lines (Tukey′s test, *P < *0.05).

**FIG 2:**
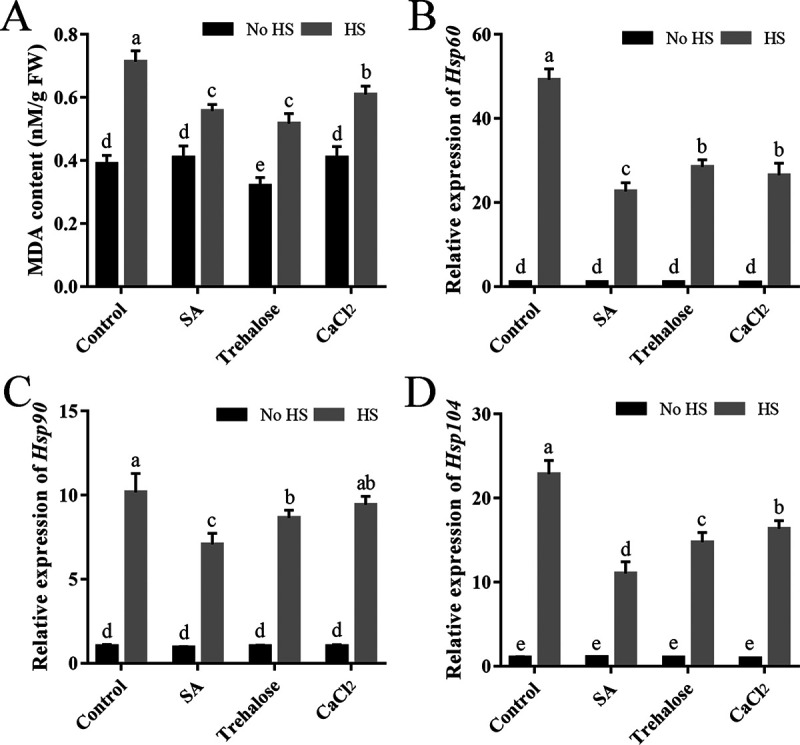
SA treatment decreased the MDA content and *HSP* expression of *P. ostreatus* under HS. The WT strain was cultured in liquid CYM supplemented with SA (200 μM) at 28°C for 3 days and then treated at 40°C for 12 h, after which the fungi were cultured at 28°C to recover for 3 days. Trehalose (25 mM) and CaCl_2_ (5 mM) were added just before HS treatment. (A) MDA content. (B to D) Relative expression levels of *Hsp60*, *Hsp90*, and *Hsp104*. The expression levels of *Hsp60*, *Hsp90*, and *Hsp104* in the WT strain were arbitrarily set to 1.0. Three independent biological replicates were performed for all experiments. The values are interpreted as the mean ± SD. The standard deviations are indicated by error bars. The small letters indicate significant differences between the lines (Tukey′s test, *P < *0.05).

Furthermore, the relative growth rates of fungal strains treated with SA under HS were detected to explore whether SA could induce fungal resistance to HS. SA treatment (200 μM) for short- or long-term treatment relieved the inhibition of HS on fungal growth (Fig. 1A). Under HS, the fungal hyphae treated with SA for 12 h grew faster (Fig. 1A), with the growth inhibition rate decreasing by 80.47% compared with that of the control (Fig. 1B). The malondialdehyde (MDA) content and expression of HSPs (*Hsp60*, *Hsp90*, and *Hsp*104) have been reported as indicators of HSR in *P. ostreatus* (10, 15). HS significantly increased the MDA content and HSP expression of the WT strain (Fig. 2). As expected, trehalose treatment (25 mM) relieved the HSR of *P. ostreatus* (Fig. 1), with the MDA content and HSP expression decreasing (Fig. 2). Similarly, the MDA content and the relative expression levels of *Hsp60*, *Hsp90*, and *Hsp104* in the WT strain treated with SA under HS were decreased by 21.96%, 53.97%, 30.37%, and 51.68%, respectively, compared with those of the control under HS (Fig. 2). These results together indicate that SA treatment promotes fungal growth and relieves the HSR of *P. ostreatus*.

### SA treatment enhanced the production of cytosolic trehalose under HS.

The WT strain formed a flavescent circle, which was more apparent with higher concentrations of SA (Fig. S1A), indicating that SA treatment may affect the metabolism of *P. ostreatus*. To explore the effect of SA on the metabolites of *P. ostreatus*, SA treatment at 200 μM was chosen for further analysis. The intracellular polysaccharide (IPS) content in the *P. ostreatus* treated with SA was increased by 36.64% compared with that of the control (Fig. S3A). Three key enzymes involved in polysaccharide biosynthesis are UDP-glucose pyrophosphorylase (Ugp), phosphoglucomutase (Pgm), and phosphoglucose isomerase (Pgi). The relative expression levels of *Ugp*, *Pgm*, and *Pgi* in *P. ostreatus* treated with SA were 2.42-, 2.25-, and 2.40-fold those of the control (Fig. S4A). However, SA treatment did not increase the IPS content of *P. ostreatus* under HS (Fig. S3A). Meanwhile, the cytosolic trehalose content in *P. ostreatus* treated with SA under HS was 180.67 mg/g dry weight (DW), which was increased by 66.26% compared with that of the control under HS (Fig. S3B). In addition, the relative expression levels of *Tps* (trehalose-6-phosphate synthase) and *Tpp* (trehalose-6-phosphate phosphatase), which are involved in trehalose biosynthesis, were 2.86- and 2.44-fold those of the control under HS (Fig. S4B). Similarly, validamycin A (neutral trehalase inhibitor) treatment (20 μM) also increased cytosolic trehalose production in *P. ostreatus* under HS (Fig. S3B). These results indicate that SA treatment induces the biosynthesis of cytosolic trehalose in *P. ostreatus* under HS.

### SA treatment reduced the intracellular ROS level but improved the cytosolic Ca^2+^ level under HS.

**FIG 3:**
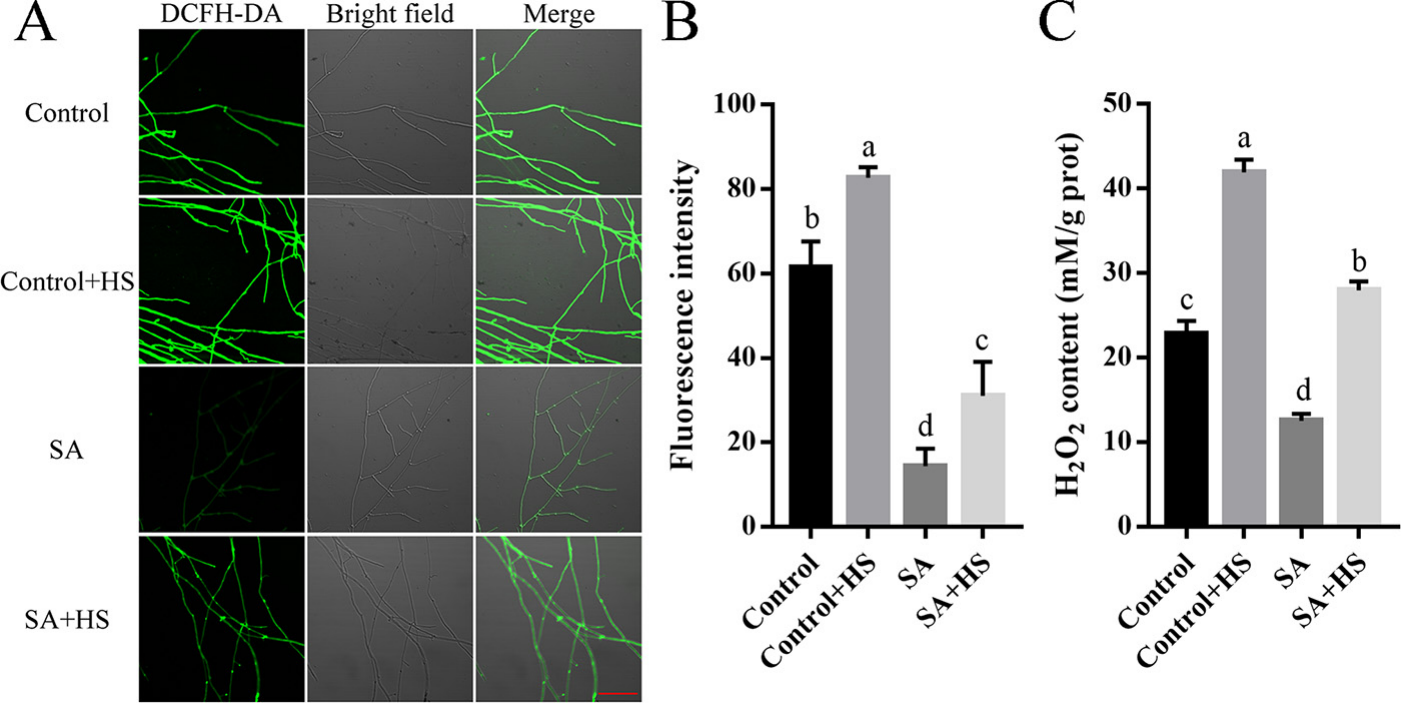
SA treatment decreased the intracellular ROS level of *P. ostreatus*. The WT strain was cultured on CYM plates (A and B) and in liquid CYM (C) supplemented with SA (200 μM) at 28°C for 3 days and then treated at 40°C for 12 h, after which the fungi were cultured at 28°C to recover for 3 days. (A) DCFH-DA staining. The fungal hyphae were stained with a fluorescent H_2_O_2_ probe (DCFH-DA), and the fluorescence was examined by a confocal laser scanning microscope with a consistent exposure time. Red scale bar = 100 μm. (B) H_2_O_2_ fluorescence value. ZEN 3.3 (blue edition) was used to analyze the fluorescence signal intensity. (C) Intracellular H_2_O_2_ levels. Three independent biological replicates were performed for all experiments. The values are interpreted as the mean ± SD. The standard deviations are indicated by error bars. The small letters indicate significant differences between the lines (Tukey′s test, *P < *0.05).

Since HS increases the intracellular ROS level (10), we first explored the effect of SA on the intracellular ROS level to understand the role of SA in fungal resistance to HS. DCFH-DA staining showed that the fluorescence intensity of H_2_O_2_ in the WT strain treated with SA under HS was significantly decreased compared with that of the control under HS (Fig. 3A). Fluorescence analysis showed that the fluorescence value in the WT strain treated with SA under HS was 31.07 and was reduced by 62.42% compared with that of the control under HS (Fig. 3B). Consistently, the H_2_O_2_ level of the WT strain treated with SA under HS was 27.99 mM/g protein and was decreased by 33.28% compared with that of the control under HS (Fig. 3C). In addition, we examined the ROS-related enzymes involved in the antioxidant system. Under HS, the APX activity of the WT strain treated with SA did not differ from that of the control (Fig. S5A). However, the activities of CAT, GPX, and SOD of the WT strain treated with SA under HS were increased by 49.52%, 57.24%, and 55.79%, respectively, compared with those of the control under HS (Fig. S5B to D). These results indicate that SA treatment reduces the intracellular ROS level of *P. ostreatus* under HS.

ROS signaling is related to Ca^2+^ signaling (21), and we examined the influence of SA on the cytosolic Ca^2+^ level. Fluo-3AM staining showed that the fluorescence intensity of cytosolic Ca^2+^ in the WT strain treated with SA under HS was significantly increased (Fig. S6A), and its fluorescence value was 2.10-fold that of the control under HS (Fig. S6B). Furthermore, the transcriptional regulation of Ca^2+^ signaling was examined in the WT strain treated with SA under HS, including Ca^2+^-permeable channel (Cch and Mid), vacuole Ca^2+^ channel (Yvc), phospholipase C (Plc), calmodulin (Cam), calcineurin (Cna1 and Cna2), calcineurin-responsive zinc finger transcription factor (Crz), and Ca^2+^/Cam-dependent protein kinase (Camk1, Camk2, and Camk3) (22). With the exceptions of *Cna2* and *Camk3*, the expression levels of Ca^2+^ signaling genes in the WT strain treated with SA under HS were upregulated compared with those in the control under HS (Fig. S6C). These results indicate that SA treatment improves the cytosolic Ca^2+^ level of *P. ostreatus* under HS.

### Effects of H_2_O_2_ addition on MDA content and *HSP* expression in fungal strains treated with SA under HS.

**FIG 4:**
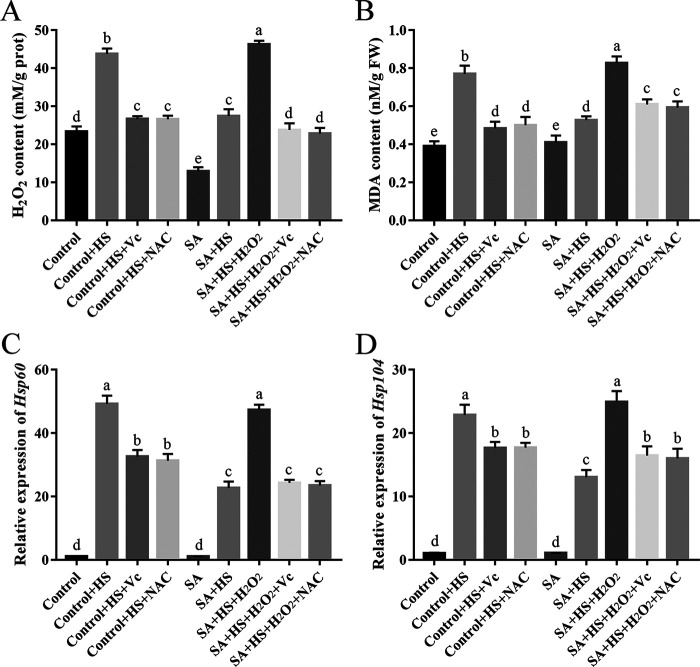
H_2_O_2_ restored the MDA content and *HSP* expression regulated by SA under HS. The WT strain was cultured in liquid CYM supplemented with SA (200 μM) at 28°C for 3 days and then treated at 40°C for 12 h, after which the fungi were cultured at 28°C to recover for 3 days. H_2_O_2_ (4 mM), Vc (2 mM), or NAC (2 mM) was added just before HS treatment. (A) Intracellular H_2_O_2_ levels. (B) MDA content. (C) Relative expression of *Hsp60*. (D) Relative expression of *Hsp104*. The expression levels of *Hsp60* and *Hsp104* in the control strain were arbitrarily set to 1.0. Three independent biological replicates were performed for all experiments. The values are interpreted as the mean ± SD. The standard deviations are indicated by error bars. The small letters indicate significant differences between the lines (Tukey′s test, *P < *0.05).

To analyze in depth the function of SA in fungal resistance to HS, we examined the role of H_2_O_2_ regulated by SA under HS. Under HS, adding 4 mM H_2_O_2_ to the WT strain treated with SA restored the decreased H_2_O_2_ content to the level of the control (Fig. 4A). In addition, we measured the MDA content and the expression levels of *Hsp60* and *Hsp104* based on the above results. Under HS, the MDA content and the expression levels of *Hsp60* and *Hsp104* in the WT strain treated with SA were reduced by 31.60%, 56.78%, and 26.00%, respectively, compared with those in the control and could be restored by 4 mM H_2_O_2_ treatment (Fig. 4B to D). Furthermore, we used ROS scavengers (Vc or NAC) to further examine the role of H_2_O_2_ induced by SA under HS. Upon adding Vc or NAC (2 mM) to the WT strain treated with SA and H_2_O_2_ under HS, the H_2_O_2_ and MDA contents and expression levels of *Hsp60* and *Hsp104* were reversed to those levels of the WT strain treated with SA (Fig. 4). These results together imply that SA treatment might decrease the MDA content and HSP expression via the intracellular ROS level in *P. ostreatus* under HS.

### Effect of inhibiting the cytosolic Ca^2+^ level on cytosolic trehalose accumulation in fungal strains treated with SA under HS.

**FIG 5:**
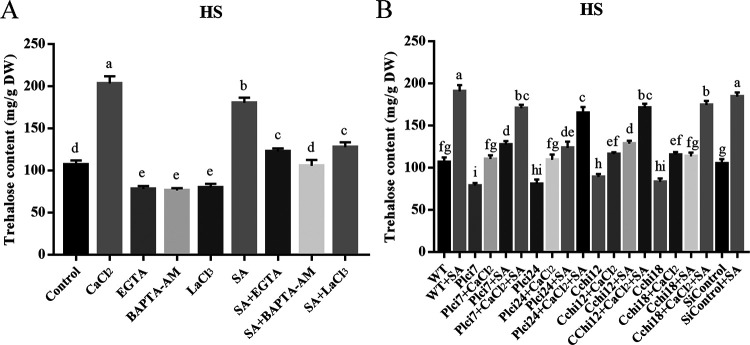
Inhibiting cytosolic Ca^2+^ levels reversed the cytosolic trehalose content induced by SA under HS. The fungal strains were cultured in liquid CYM supplemented with SA (200 μM) at 28°C for 3 days and then treated at 40°C for 12 h, after which the fungi were cultured at 28°C to recover for 3 days. CaCl_2_ (5 mM) and Ca^2+^ inhibitors (EGTA, 5 mM; BAPTA-AM, 1 mM; LaCl_3_, 5 mM) were added just before HS treatment. (A) Effect of Ca^2+^ inhibitors on trehalose accumulation in fungal strains. (B) Effect of silencing *Plc* or *Cch* on trehalose accumulation in fungal strains. Three independent biological replicates were performed for all experiments. The values are interpreted as the mean ± SD. The standard deviations are indicated by error bars. The small letters indicate significant differences between the lines (Tukey′s test, *P < *0.05).

Trehalose accumulation in *P. ostreatus* under HS is induced by intracellular ROS (10). However, we observed that SA treatment reduced the intracellular ROS level but increased cytosolic trehalose accumulation and the cytosolic Ca^2+^ level under HS, implying that the cytosolic Ca^2+^ level might play a role in cytosolic trehalose accumulation to relieve HSR in *P. ostreatus* treated with SA. To verify our hypothesis, CaCl_2_ and Ca^2+^ inhibitors, including EGTA (Ca^2+^ chelator), BAPTA-AM (intracellular Ca^2+^ chelator), and LaCl_3_ (plasma membrane Ca^2+^ channel blocker), were used. As expected, CaCl_2_ (5 mM) treatment induced fungal resistance to HS (Fig. 1), with the MDA content and *HSP* expression decreased (Fig. 2). Furthermore, adding CaCl_2_ to the WT strain under HS increased the cytosolic trehalose content by 90.41% compared with that of the control under HS (Fig. 5A). Meanwhile, the cytosolic trehalose contents of the WT strain treated with SA plus 5 mM EGTA, 1 mM BAPTA-AM, or 5 mM LaCl_3_ under HS were 122.75, 105.51, and 127.83 mg/g DW and were reduced by 31.93%, 41.48%, and 29.11%, respectively, compared with that of the WT strain treated with SA under HS (Fig. 5A). These results indicate that CaCl_2_ treatment induces fungal resistance to HS, and Ca^2+^ inhibitors could reduce the improved trehalose accumulation induced by SA treatment in *P. ostreatus* under HS.

The *Plc* gene is reported to regulate Ca^2+^ release from intracellular pools (23), and the *Cch* gene can mediate Ca^2+^ entry from extracellular stores (24). To confirm the role of the cytosolic Ca^2+^ level regulated by SA on trehalose accumulation, silenced strains for *Plc* and *Cch* were constructed. Plc (GenBank: ON229075) and Cch (GenBank: ON229076) were obtained by BLAST analysis against the genome of P89 using Plc (GenBank: KAF7439746) and Cch (GenBank: XP_036625629) of *P. ostreatus* PC9, respectively. The antisense fragments of *Plc* and *Cch* were used to construct the silencing vectors RNAi-PLC and RNAi-CCH (Fig. S7B to C), respectively, with pHPG containing the *hyg* gene as a selectable marker. The silencing efficiencies of *Plc* in Plci7 and Plci24 were 65.00% and 59.33% (Fig. S8A), and the silencing efficiencies of *Cch* in Cchi12 and Cchi18 were 75.67% and 78.33% (Fig. S8B), respectively. Under HS, the cytosolic trehalose contents of Plci7, Plci24, Cchi12, and Cchi18 were significantly decreased compared with that of the WT strain and could be restored by CaCl_2_ or SA treatment (Fig. 5B). In addition, the trehalose contents in these silenced strains treated with SA and CaCl_2_ under HS were reversed to those levels of the WT strain treated with SA under HS (Fig. 5B). These results indicate that silencing *Plc* or *Cch* could reduce the improved trehalose accumulation induced by SA treatment in *P. ostreatus* under HS.

### Effects of silencing *Tps* on MDA content and *HSP* expression in fungal strains treated with SA under HS.

**FIG 6:**
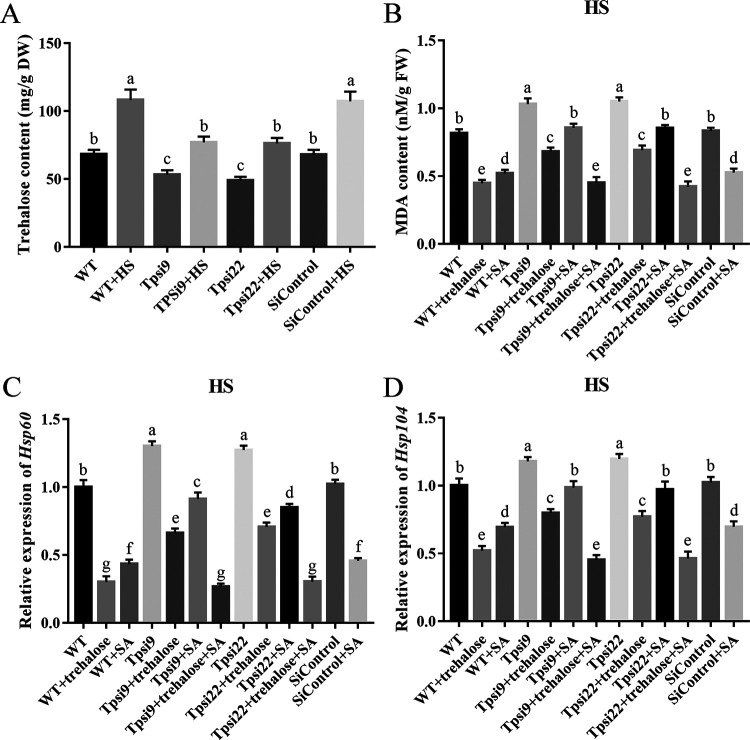
Silencing *Tps* restored the MDA content and HSP expression regulated by SA under HS. The fungal strains were cultured in liquid CYM supplemented with SA (200 μM) at 28°C for 3 days and then treated at 40°C for 12 h, after which the fungi were cultured at 28°C to recover for 3 days. Trehalose (25 mM) was added just before HS treatment. (A) Cytosolic trehalose content. (B) MDA content. (C) Relative expression of *Hsp60*. (D) Relative expression of *Hsp104*. The expression levels of *Hsp60* and *Hsp104* in the WT strain under HS were arbitrarily set to 1.0. Three independent biological replicates were performed for all experiments. The values are interpreted as the mean ± SD. The standard deviations are indicated by error bars. The small letters indicate significant differences between the lines (Tukey′s test, *P < *0.05).

The Tps (GenBank: AWX94602) of *P. ostreatus* is reported to catalyze the first step of trehalose synthesis (25). To understand the role of cytosolic trehalose induced by SA on HS resistance in detail, silencing vectors for *Tps* were constructed (Fig. S7D). The silencing efficiencies of *Tps* in *Tps*-silenced strains (Tpsi9 and Tpsi22) were 78.00% and 72.67%, respectively (Fig. S8C). As expected, the cytosolic trehalose contents in Tpsi9 and Tpsi22 under HS were decreased by 28.92% and 29.54% compared with that of the WT strain under HS (Fig. 6A), respectively. Under HS, the MDA contents and *HSP* (*Hsp60* and *Hsp104*) expression levels in Tpsi9 and Tpsi22 were significantly increased compared with those of the WT strain and could be reversed by trehalose (25 mM) or SA treatment (Fig. 6B to D), respectively. In addition, the MDA contents and *HSP* expression levels in Tpsi9 and Tpsi22 treated with SA under HS were significantly increased compared with those of the WT strain treated with SA under HS and could be restored by trehalose treatment (Fig. 6B to D). These results together indicate that silencing *Tps* could inhibit trehalose biosynthesis to restore the decreased MDA content and the downregulated *HSP* expression regulated by SA treatment in *P. ostreatus* under HS.

## DISCUSSION

SA has been recognized as a plant hormone that mainly mediates plant defenses against pathogens and functions well in plant responses to numerous abiotic stresses, such as drought, chilling, and heat (26). The crucial roles of SA in plant morphogenesis have also been confirmed. However, the roles of SA in filamentous fungi, especially in basidiomycetes, are not well understood. Previous studies showed that acetylsalicylic acid was increased in the extracellular fluid of *P. ostreatus* exposed to high temperature (14), and the content of cellular SA was significantly increased (29-fold) in heat-treated *P. ostreatus* (20), indicating that SA might play an important role in the HSR of *P. ostreatus*. Illustrating the function of SA on the HSR of *P. ostreatus* is important for its cultivation. Here, we explored the roles of exogenous SA in *P. ostreatus*. Our results showed that SA treatment improved fungal growth and increased the accumulation of cytosolic trehalose in *P. ostreatus*. Furthermore, SA treatment could mediate the HSR of *P. ostreatus* through the intracellular ROS level and cytosolic trehalose content.

Numerous studies have confirmed the key roles of SA in various physiological processes of plants. As reported, SA is the key plant defense hormone required for resistance to many microbial pathogens (18). Despite its significant functions in disease resistance, the remarkable effect of SA on the physiological phenotypes of plants has been well established (26, 27). The effects of SA on the length of the radicle or primary root are concentration-dependent in plants. In pearl millet, 0.5 mM SA treatment causes increased root length in one variety, whereas SA treatment (0.5–3 mM) decreased the root length in another variety (28). In filamentous fungi, the phytohormones ethylene (29) and methyl jasmonate (30) are reported to have promotion effects on the fungal growth of Ganoderma lucidum, indicating that phytohormones might increase fungal resistance to heat stress. Here, we found that the effect of SA on fungal growth was concentration dependent in *P. ostreatus*. SA treatment at low concentrations promoted fungal growth, while higher concentrations of SA treatment decreased fungal growth (Fig. S1). The effect of SA treatment on fungal resistance to heat stress was also concentration dependent (data not shown). Moreover, SA treatment at 200 μM increased the fungal resistance to heat stress in *P. ostreatus*. In our experiment, the minimal SA concentration to have an obvious heat-protective effect was approximately 10 μM, which was similar to a previous report (31). It has been reported that cytosolic calcium dynamics are related to hyphal and colony growth in the filamentous fungus *Colletotrichum graminicola* (32). Consistently, the cytosolic Ca^2+^ level was improved in *P. ostreatus* treated with SA (Fig. S6), indicating that SA treatment might regulate fungal growth via the cytosolic Ca^2+^ level in *P. ostreatus*. In addition, SA can be used in all stages of *P. ostreatus* cultivation, while calcium is more appropriate for use in the compost of *P. ostreatus* (33).

The enhancing effects of SA on the production of secondary metabolites were reported in plants. SA treatment increased the production of artemisinin in *Artemisia annua* under arsenic stress (34) and induced the accumulation of sucrose in peach fruit (35). In filamentous fungi, the application of 200 μM SA increased the accumulation of ganoderic acids in Ganoderma lucidum (36), and SA could inhibit complex III activity to produce ROS and thereby induce ganoderic acid overproduction (37). Consistently, SA treatment at 200 μM improved the production of IPS and cytosolic trehalose in *P. ostreatus* (Fig. S3). Moreover, SA treatment elevated the cytosolic Ca^2+^ level (Fig. S6), and the addition of Ca^2+^ inhibitors restored the improved trehalose content induced by SA treatment in *P. ostreatus* under HS (Fig. 4). These results imply that the cytosolic Ca^2+^ level is involved in trehalose biosynthesis regulated by SA treatment in *P. ostreatus*.

HS is an abiotic environmental stress that seriously affects the growth and development of microorganisms. As reported, HS disrupts cell wall integrity (8) and changes the metabolites of *P. ostreatus* (20). Consistent with previous findings (10, 15), the MDA content was significantly increased, and *HSP* expression was upregulated with high amplitude in *P. ostreatus* treated with HS (Fig. 1). Therefore, MDA content and *HSP* expression were used as indicators of HSR. Here, SA treatment was found to induce fungal resistance to HS in *P. ostreatus* (Fig. 1), which is similar to the functions of SA in plants (38). The intracellular ROS level was increased in *P. ostreatus* under HS, indicating that the MDA level was positively correlated with ROS accumulation as reported previously (10). It was reported that the accumulation of H_2_O_2_ and lipid peroxidation (MDA content) could result in a significant decrease in cell membrane stability, reflecting the extent of lipid peroxidation caused by ROS (39). Moreover, we found that SA treatment could reduce the increased intracellular H_2_O_2_ content induced by HS, with decreases in the MDA content and *HSP* expression (Fig. 4). In addition, H_2_O_2_ could restore the intracellular H_2_O_2_ and MDA contents and *HSP* expression of the WT strain treated with SA under HS (Fig. 4). Together, our results imply that SA treatment can alleviate the HSR of *P. ostreatus* via the intracellular ROS level.

The nonreducing disaccharide trehalose plays a crucial role in tolerance to numerous stresses. In Pseudomonas aeruginosa, trehalose synthesis mutants displayed seriously compromised growth under salt stress (40). Exogenous trehalose is reported to play a role in the responses of wheat roots exposed to high temperature (41). Similarly, trehalose can regulate the HSR of *P. ostreatus* by influencing central carbon metabolism and thus alleviating high-temperature stress (16). Here, we showed that trehalose or CaCl_2_ treatment induced fungal resistance to HS, and SA treatment increased the cytosolic trehalose content (Fig. 5A) and the cytosolic Ca^2+^ levels (Fig. S6) of *P. ostreatus* under HS. In addition, CaCl_2_ treatment increased the cytosolic trehalose content of *P. ostreatus* under HS (Fig. 5). Moreover, we found that inhibiting the cytosolic Ca^2+^ level using Ca^2+^ inhibitors or mutants (*Plc*-silenced and *Cch*-silenced strains) reversed the increased trehalose content induced by SA treatment in *P. ostreatus* under HS (Fig. 5). These results indicate that SA treatment might regulate trehalose biosynthesis via cytosolic Ca^2+^ levels. In addition, we constructed *Tps*-silenced strains to inhibit trehalose biosynthesis (Fig. 6A). The decreased MDA content and *HSP* expression in the WT strain treated with SA under HS were restored in *Tps*-silenced strains treated with SA under HS (Fig. 6B to D), implying that the trehalose biosynthesis induced by SA treatment can alleviate the HSR of *P. ostreatus*. This is consistent with the role of trehalose in HS reported previously (10). Together, our findings indicate that SA treatment can induce cytosolic trehalose biosynthesis via the intracellular Ca^2+^ level and thus alleviate the HSR of *P. ostreatus*.

**FIG 7:**
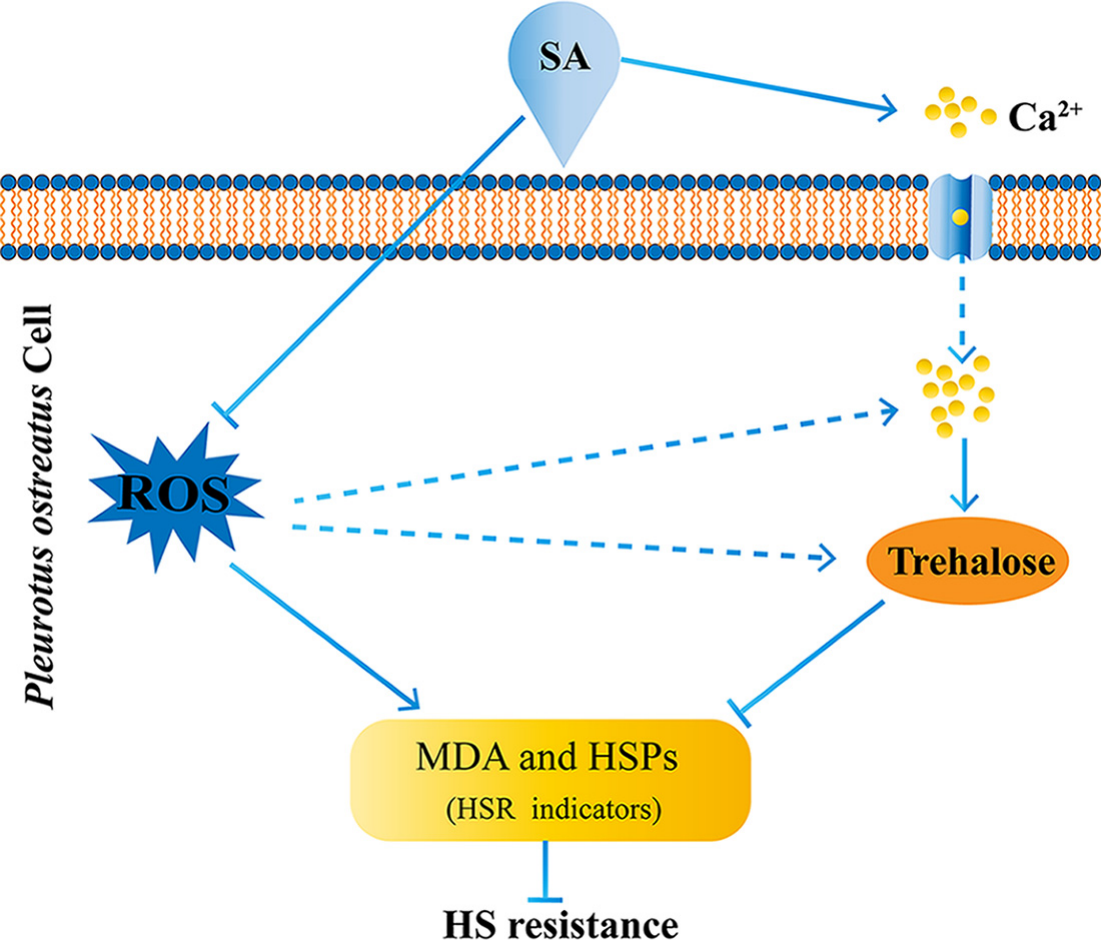
Schematic representation of the potential cascade of cellular components constituting the SA-mediated HSR in *P. ostreatus*. SA treatment induces fungal resistance to HS. On the one hand, SA treatment inhibits the intracellular ROS level to affect HSR indicators (the intracellular MDA content and *HSP* expression). On the other hand, SA treatment induces cytosolic trehalose biosynthesis via cytosolic Ca^2+^ levels and thus affects HSR indicators. The solid arrows represent the data supported by our experiments, and the dotted arrows indicate data supported in previous studies or other fungal systems.

In summary, SA treatment improved fungal growth and cytosolic trehalose accumulation in *P. ostreatus* under HS. In addition, SA treatment decreased the intracellular ROS level but elevated the cytosolic Ca^2+^ level of *P. ostreatus* under HS. Further analysis showed that the intracellular ROS level and cytosolic trehalose content regulated by SA treatment were involved in the HSR of *P. ostreatus*. SA treatment can alleviate HSR in *P. ostreatus* by reducing the intracellular ROS level and increasing trehalose biosynthesis via the cytosolic Ca^2+^ level. Based on our findings, the cascade of the cellular components constituting the SA-mediated HSR of *P. ostreatus* was proposed (Fig. 7). Our study will help to understand the mechanism of SA-mediated HSR in filamentous fungi and provide new insights for the cultivation of *P. ostreatus*.

## MATERIALS AND METHODS

### Microbial strains and culture conditions.

The *P. ostreatus* strain (CCMSSC 00389, P89) was obtained from the Agricultural Culture Collection of China (ACCC50596) and used as a wild-type (WT) strain for constructing gene-silenced strains. The formulation of the culture medium and the fungal growth conditions were conducted as previously described (10) with slight modifications. Briefly, the fungal strains were maintained on potato dextrose agar (PDA) plates at 28°C. For fungal growth tests and recovery growth tests under HS, 6-mm-diameter hyphal tip plugs from PDA plates were inoculated onto complete yeast medium (CYM) plates (16) with or without other substances and cultured at 28°C for 7 days. For fermentation experiments, 6-mm-diameter hyphal tip plugs were inoculated into 100 mL of potato dextrose broth (PDB) to prepare seed cultures. Then, 4 mL of seed culture was inoculated into liquid CYM and cultured at 28°C and 150 rpm for 7 days. Escherichia coli DH5α (TaKaRa, Dalian, China) and Agrobacterium tumefaciens GV3101 (IMCAS, Beijing, China) were grown in lysogeny broth with or without ampicillin, kanamycin, or rifampin as reported previously (9).

### HS treatment.

The HS and recovery treatments of fungal strains were performed as described elsewhere (10) with slight modifications. Briefly, the fungal strains were incubated on CYM plates or liquid CYM at 28°C for 3 days and then treated at 40°C for the indicated time, followed by returning to culture at 28°C to recover for 3 days.

### Construction of RNAi plasmids and strains.

Gene silencing is effective for exploring the roles of fungal genes. In the present study, gene silencing plasmids were constructed using the pBHG vector (42). The sense and antisense sequences of the glyceraldehyde-3-phosphate dehydrogenase (gpd) promoter (GenBank: KY924471) of *P. ostreatus* were amplified by PCR and digested and were then inserted into pBHG at the corresponding restriction sites to generate the plasmid pHPG (Fig. S7A). The antisense fragments of the target gene (*Plc*, *Cch*, and *Tps*) were amplified by PCR using *P. ostreatus* cDNA as the template. Then, the obtained fragments were double digested with *Bgl*II and ApaI and inserted into pHPG to generate the RNAi plasmids (RNAi-PLC, RNAi-CCH, and RNAi-TPS). The constructed plasmid maps of the RNAi plasmids are shown in Fig. S7. Then, the RNAi plasmids were transferred into *P. ostreatus* with A. tumefaciens-mediated transformation as reported previously (43). The obtained transformants were selected on CYM containing 90 μg/mL hygromycin B (hyg). Two independent silencing strains of each target gene with higher silencing efficiency were selected for further study. The primers used are listed in Table S1.

### Determining the length between hyphal branches.

To determine the length between hyphal branches, the fungal strains were cultured on CYM plates with or without SA at 28°C, and sterilized coverslips were inserted into the CYM plates as reported previously (44). The length between hyphal branches was analyzed as described elsewhere (45).

### Enzymatic activity determination.

To detect the enzymatic activity of the antioxidant systems, the fungal strains were cultured in liquid CYM under the indicated conditions, and the mycelia were collected. Crude protein was prepared, and the enzymatic activities of superoxide dismutase (SOD), catalase (CAT), glutathione peroxidase (GPX), and ascorbate peroxidase (APX) were measured as previously described (10).

### Detection of the malondialdehyde content.

The mycelia of fungal strains were collected from liquid CYM cultures to measure the malondialdehyde (MDA) content. The MDA level was determined as described previously (10).

### Measurement of the intracellular ROS level.

For the intracellular ROS fluorescence assay, the fungal hyphae on coverslips from CYM plates were stained with 2′,7′-dichlorodihydrofluorescein diacetate (DCFH-DA) to visualize H_2_O_2_ by an LSM780 confocal laser scanning microscope (Zeiss, Jena, Germany) as described previously (44). In addition, the fungal hyphae collected from liquid CYM were used to measure the intracellular H_2_O_2_ content by a commercial hydrogen peroxide assay kit (Nanjing Jiancheng Bioengineering Institute, Nanjing, China) as described previously (44).

### Detection of the cytosolic Ca^2+^ level.

Fluo-3AM is an acetoxymethyl ester of a fluorescent calcium indicator dye. This dye is readily hydrolyzed into a Ca^2+^-binding form Fluo-3 by an endogenous esterase after it enters a cell. To detect the cytosolic Ca^2+^ level, the fungal hyphae on coverslips from CYM plates were stained with Fluo-3AM and examined using an LSM780 confocal laser scanning microscope (Zeiss, Jena, Germany) as reported previously (46).

### Determination of IPS and cytosolic trehalose contents.

To detect the IPS content, the fungal mycelia were collected from liquid CYM cultures and repeatedly washed using distilled water followed by drying at 60°C. The IPS was extracted from dried mycelia with 1 M NaOH at 60°C for 1 h as previously reported (47). After centrifugation at 4,000 × *g* for 5 min, the supernatant was analyzed by the phenol-sulfuric acid method (48). The intracellular trehalose content was measured using the method described elsewhere (25).

### Analysis of gene expression by qRT–PCR.

For the analysis of gene-specific mRNAs expressed by the WT and RNAi strains, the fungal strains were cultured on CYM plates or in liquid CYM under the indicated conditions. Total RNA was extracted, and the first-strand cDNAs of fungal strains were synthesized as previously described (44). Quantitative real-time PCR (qRT–PCR) for the detection of gene-specific mRNA levels was performed with a Realplex2 System (Eppendorf, Hamburg, Germany) using EvaGreen 2× qPCR MasterMix-S (ABM, Richmond, Canada) as described in a previous study (44). The glyceraldehyde-3-phosphate dehydrogenase gene (*GAPDH*) was chosen as a standard control (16). The post-qRT–PCR calculations to analyze the relative gene-specific mRNA expression levels were performed with the 2^-△△CT^ method (49). The primers used for qRT–PCR are listed in Table S1. The GenBank accession numbers for the genes used are listed in Table S2.

### Statistical analysis.

All of the experiments in the present study were carried out in at least three replicates with similar results. GraphPad Prism version 7.00 for Windows (GraphPad Software, Inc., La Jolla CA, USA; www.graphpad.com) was used for the statistical analysis. The results are interpreted as the mean ± standard deviation (SD). Error bars represent the SDs from the means of triplicates. One-way ANOVA followed by Tukey′s honest significant difference test (*P < *0.05) was used for the significance analysis between the analyzed samples.
