# Supplementary material for: Salicylic Acid Treatment Alleviates the Heat Stress Response by Reducing the Intracellular ROS Level and Increasing the Cytosolic Trehalose Content in Pleurotus ostreatus
Source: Microbiol Spectr. 2022 Dec 12;11(1):e03113-22. doi: 10.1128/spectrum.03113-22 (PMC9927586; doi:10.1128/spectrum.03113-22)
Supplement: Supplemental file 1 — Supplemental material. Download spectrum.03113-22-s0001.pdf, PDF file, 1.1 MB [file spectrum.03113-22-s0001.pdf]

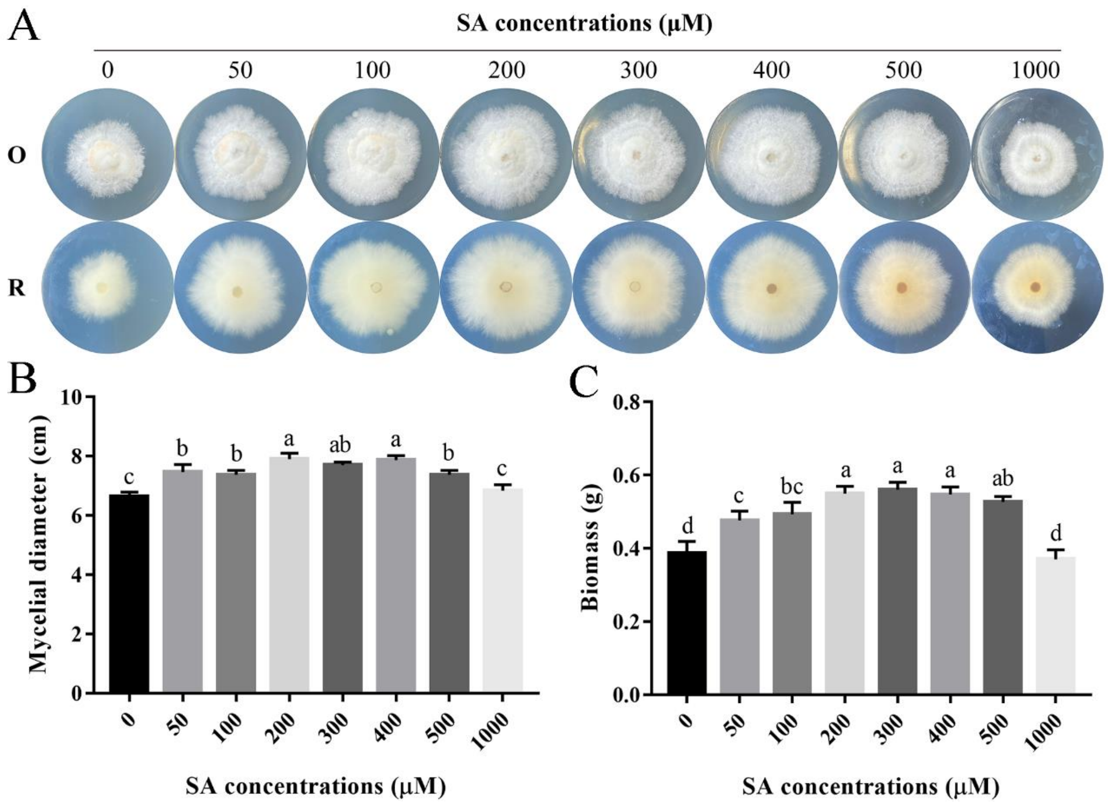

FIG S1 Effect of SA treatments on fungal growth. (A) Fungal growth of *P. ostreatus* treated with SA. The WT strain was cultured on CYM plates supplemented with different concentrations of SA at 28 °C for 5 days. The obverse sides (O) and reverse sides (R) of the plates were photographed. (B) Mycelial diameter analysis. (C) Fungal biomass. The WT strain was cultured in liquid CYM supplemented with different concentrations of SA at 28 °C for 7 days. Three independent biological replicates were performed for all experiments. The values are interpreted as the mean ± SD. The standard deviations are indicated by error bars. The small letters indicate significant differences between the lines (Tukey′s test, $P < 0.05$).

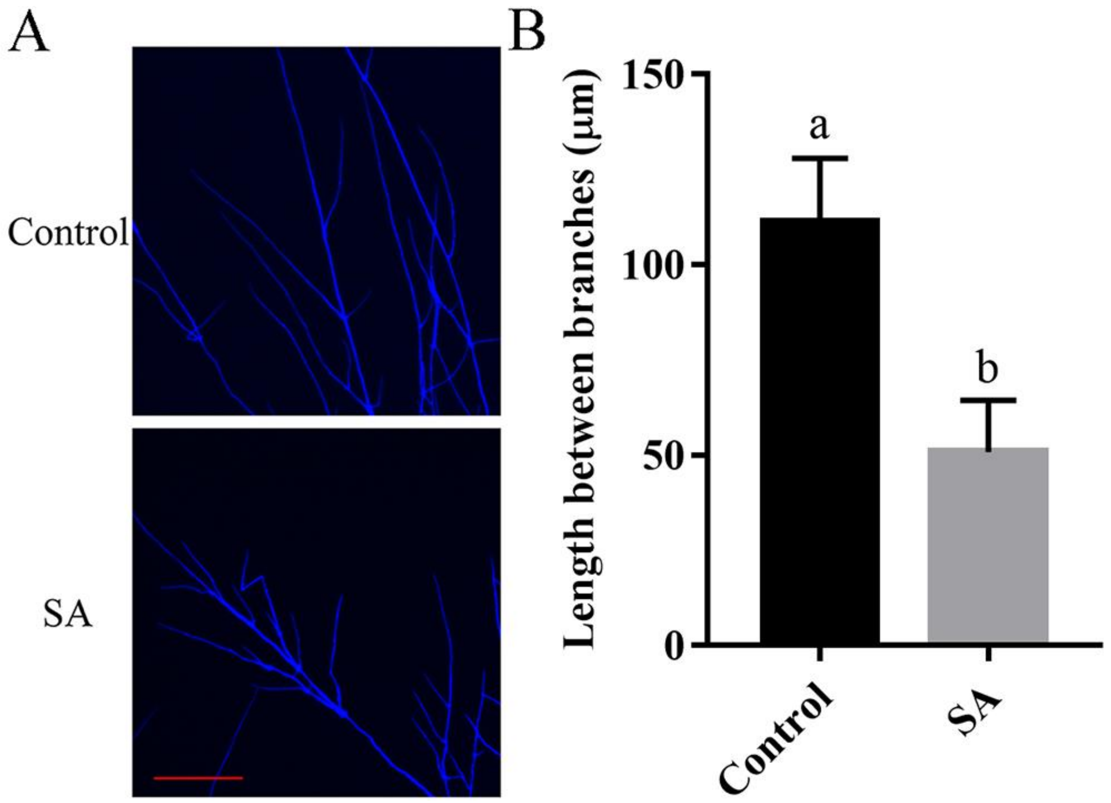

FIG S2 Effect of SA treatment on hyphal branching. The WT strain was cultured in CYM plates supplemented with SA (200 μM) at 28 °C. (A) Hyphal branching of the fungal strains (scale bar = 100 μm). (B) Length between hyphal branches in the fungal strains. Three independent biological replicates were performed for all experiments. The values are interpreted as the mean ± SD. The standard deviations are indicated by error bars. The small letters indicate significant differences between the lines (Tukey′s test, $P < 0.05$).

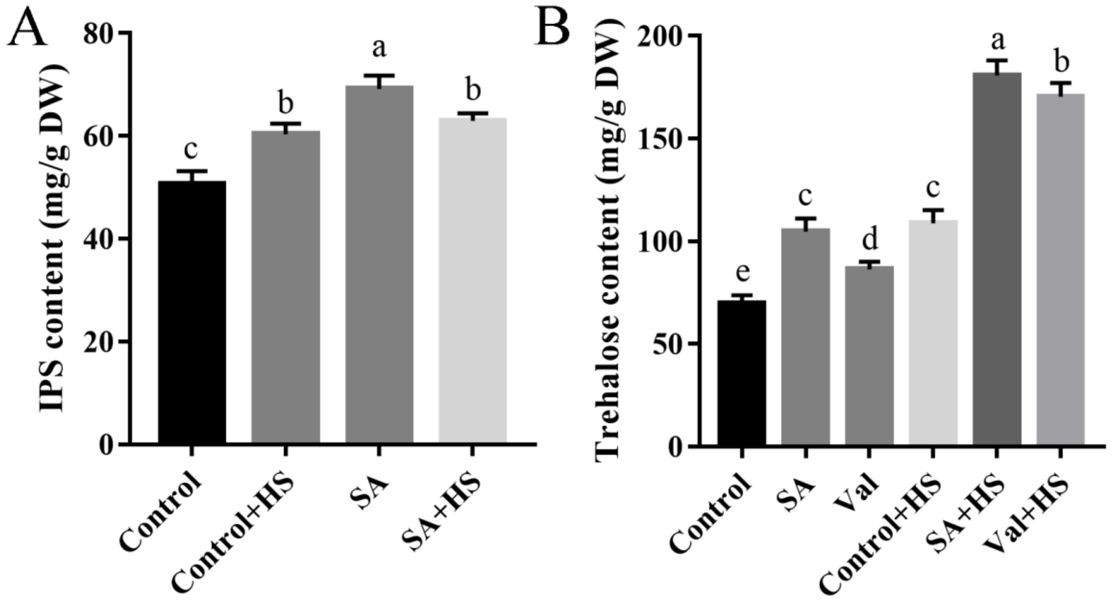

FIG S3 Effects of SA treatment on the metabolites of *P. ostreatus*. The WT strain was cultured in liquid CYM supplemented with SA (200 μM) at 28 °C for 3 days and then treated at 40 °C for 12 h, after which the fungi were cultured at 28 °C to recover for 3 days. The neutral trehalase inhibitor validamycin A (Val) was added just before HS treatment. (A) Intracellular polysaccharide (IPS) content. (B) Cytosolic trehalose content. Three independent biological replicates were performed for all experiments. The values are interpreted as the mean ± SD. The standard deviations are indicated by error bars. The small letters indicate significant differences between the lines (Tukey′s test, $P < 0.05$).

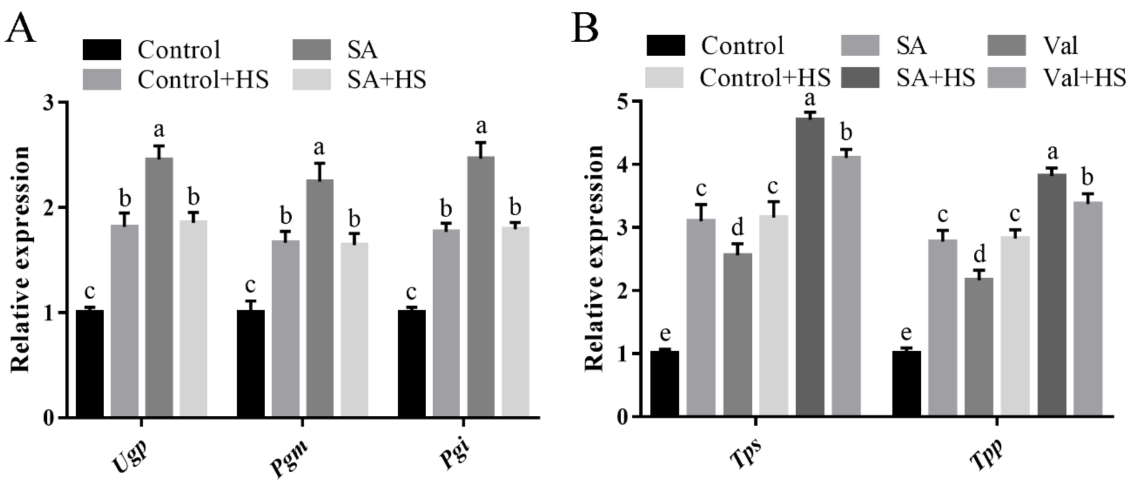

FIG S4 Effects of SA treatment on the expression of polysaccharide- and trehalose-related genes. The WT strain was cultured in liquid CYM supplemented with SA (200 μM) at 28 °C for 3 days and then treated at 40 °C for 12 h, after which the fungi were cultured at 28 °C to recover for 3 days. The neutral trehalase inhibitor validamycin A (Val, 20 μM) was added just before HS treatment. (A) Relative expression levels of polysaccharide-related genes. (B) Relative expression levels of trehalose-related genes. The expression levels of *Ugp*, *Pgm*, *Pgi*, *Tps* and *Tpp* in the control strain were arbitrarily set to 1.0. Three independent biological replicates were performed for all experiments. The values are interpreted as the mean ± SD. The standard deviations are indicated by error bars. The small letters indicate significant differences between the lines (Tukey′s test, $P < 0.05$).

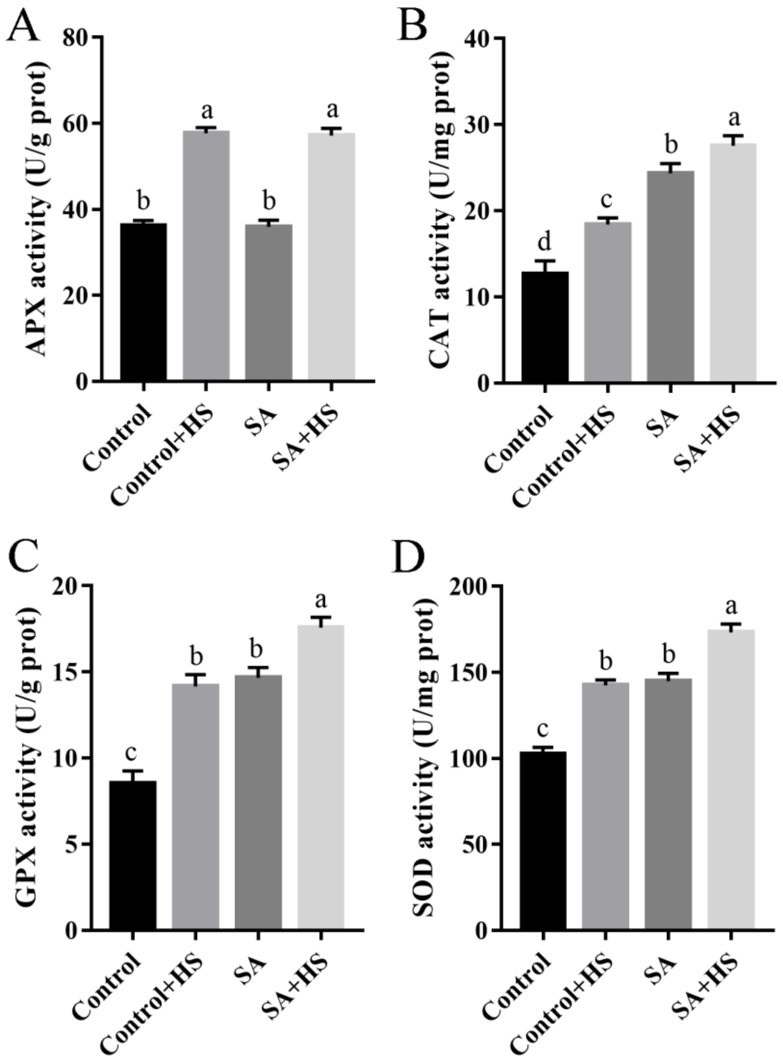

FIG S5 Activities of ROS-related enzymes involved in the antioxidant system in fungal strains. The WT strain was cultured in liquid CYM supplemented with SA (200 μM) at 28 °C for 3 days and then treated at 40 °C for 12 h, after which the fungi were cultured at 28 °C to recover for 3 days. (A) Enzymatic activity of APX. (B) Enzymatic activity of CAT. (C) Enzymatic activity of GPX. (D) Enzymatic activity of SOD. Three independent biological replicates were performed for all experiments. The values are interpreted as the mean ± SD. The standard deviations are indicated by error bars. The small letters indicate significant differences between the lines (Tukey′s test, $P < 0.05$).

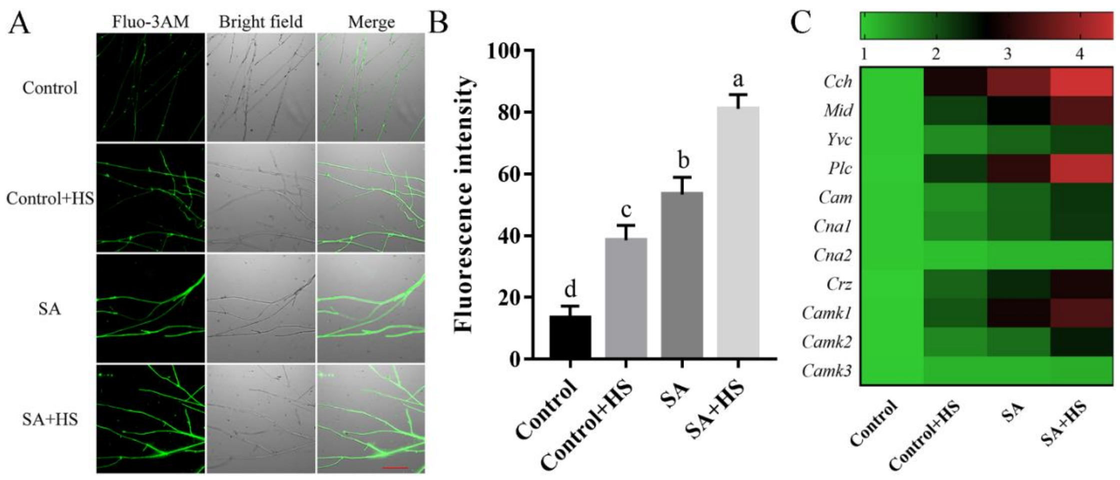

FIG S6 SA treatment elevated the cytosolic Ca^{2+} level of *P. ostreatus*. The WT strain was cultured on CYM plates supplemented with SA (200 μM) at 28 °C for 3 days and then treated at 40 °C for 12 h, after which the fungi were cultured at 28 °C to recover for 3 days. (A) Fluo-3AM staining. Fluo-3 AM is a membrane-permeable compound that can detect the level and localization of free cytosolic Ca^{2+}. The fluorescence was examined by a confocal laser scanning microscope with a consistent exposure time. Red scale bar = 100 μm. (B) Ca^{2+} fluorescence values. ZEN 3.3 (blue edition) was used to analyze the fluorescence signal intensity. (C) Transcriptional analysis of Ca^{2+}-related genes. The expression levels of Ca^{2+}-related genes in the control strain were arbitrarily set to 1.0. Three independent biological replicates were performed for all experiments. The values are interpreted as the mean ± SD. The standard deviations are indicated by error bars. The small letters indicate significant differences between the lines (Tukey′s test, $P < 0.05$).

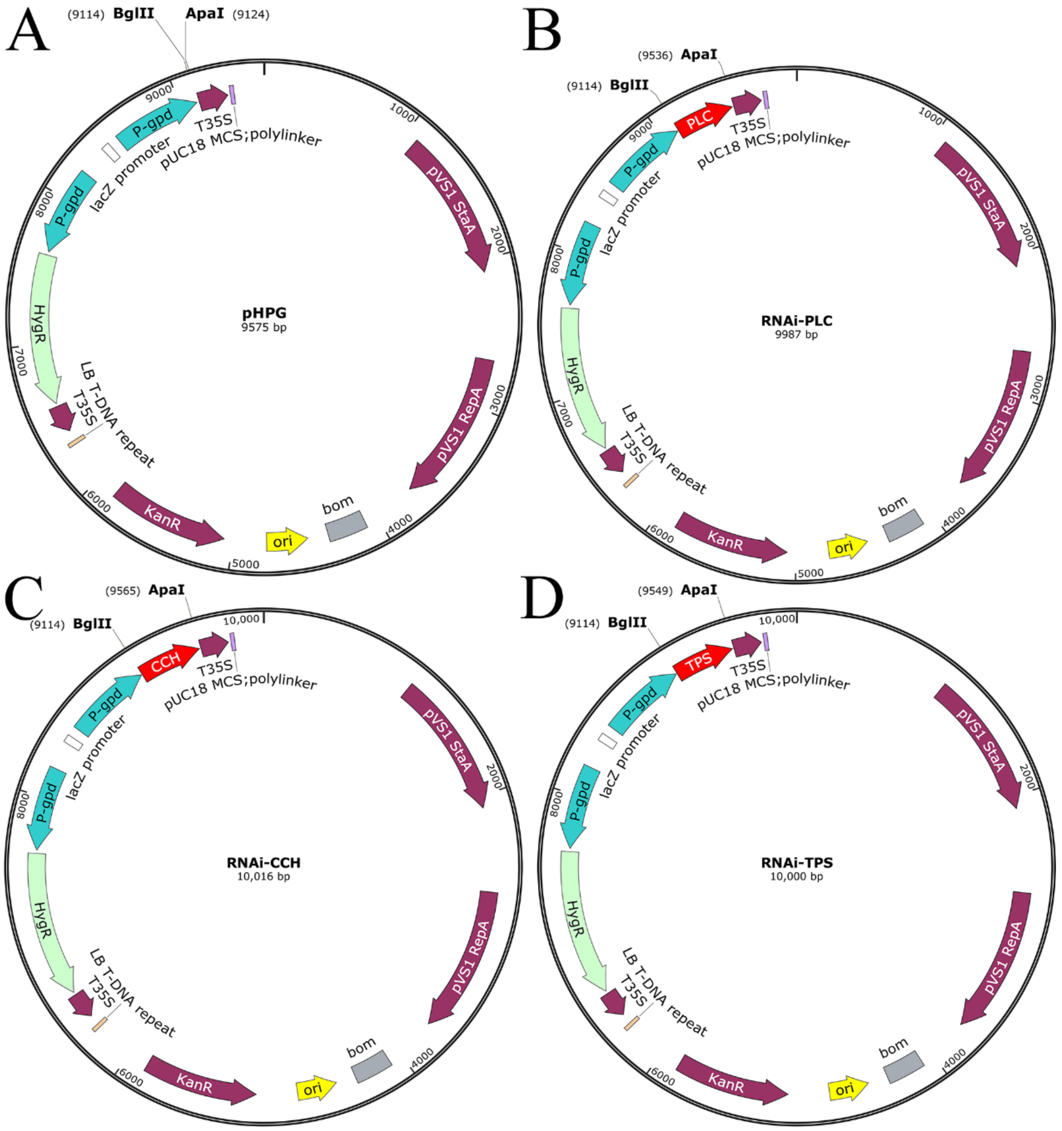

FIG S7 Structures of gene-silenced vectors. (A) Plasmid structure of pHPG. In the plasmid, the *HygR* gene is driven by the glyceraldehyde-3-phosphate dehydrogenase promoter of *P. ostreatus* (P-gpd). (B) Plasmid structure of RNAi-PLC. The 412 bp antisense fragments of *Plc* were used to silence the expression of *Plc*. (C) Plasmid structure of RNAi-CCH. The 441 bp antisense fragments of *Cch* were used to silence the expression of *Cch*. (D) Plasmid structure of RNAi-TPS. The 425 bp antisense fragments of *Tps1* were used to silence the expression of *Tps1*. The antisense fragments of *Plc*, *Cch* and *Tps* were inserted into the pHPG plasmid at the restriction enzyme sites *Bgl*II and *Apa*I and were driven by the P-gpd promoter.

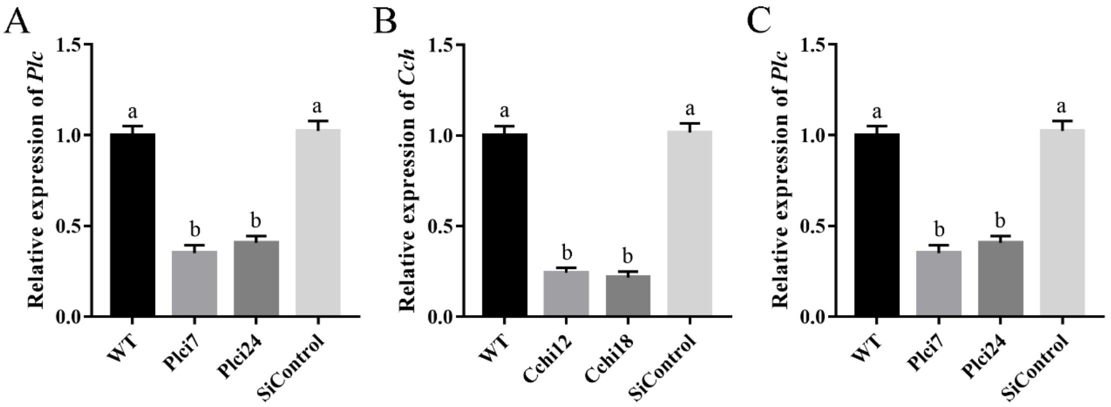

FIG S8 Silencing efficiency in the gene-silenced strains. (A) qRT–PCR analysis of the expression of *Plc* in the tested strains. (B) qRT–PCR analysis of the expression of *Cch* in the tested strains. (C) qRT–PCR analysis of the expression of *Tps* in the tested strains. The relative mRNA levels of *Plc*, *Cch* and *Tps* were calculated as the ratio of their mRNA to *GAPDH* mRNA. The expression levels of *Plc*, *Cch* and *Tps* in the WT strain were arbitrarily set to 1. The transformant used the empty vector pHPG for transformation was used as the SiControl. Three independent biological replicates were performed for all experiments. The values are interpreted as the mean ± SD. The standard deviations are indicated by error bars. The small letters indicate significant differences between the lines (Tukey′s test, $P < 0.05$).

Table S1 Oligonucleotide primers used

Primer	Sequence (5′to 3′)	Description
S-gpd-F	ATCggatccTTTATTGGCGGTTCTGA	Get the sense fragments of *gpd* of *P.*
S-gpd-R	AgggcccagatctAAGAGTGGAGATTGAGAG	*ostrestus*
AS-gpd-F	CCCaagcttAAGAGTGGAGATTGAGAG	Get the antisense fragments of *gpd*
AS-gpd-R	CCCaagcttTTTATTGGCGGTTCTGAC	of *P. ostrestus*
Plc-F	GGAagatctTGCCACGACAAGAACAG	Get the antisense silencing fragment
Plc-R	AgggcccAGCCTCCATAGACTCCC	of *Plc* gene
Cch-F	GGAagatctCGCACGCTAGTAATGAT	Get the antisense silencing fragment
Cch-R	AgggcccAGACGAGGAGGAGGAGA	of *Cch* gene
Tps-F	GGAagatctTAAGGAGTGAGTCGTGGGA	Get the antisense silencing fragment
Tps-R	AgggcccGCCGCAGGTAAAGGTGT	of *Tps* gene
GAPDH-qF	GTGTTAACCTCGAGACTTACG	Detects the *GAPDH* expression
GAPDH-qR	TGGTGGCGTGGATTGTGCTC	
Hsp60-qF	TGACGGAACAACAACAGC	Detects the *Hsp60* expression
Hsp60-qR	TTTTGGCATTAGCAGACA	
Hsp90-qF	TTACCAACGACTGGGAGGA	Detects the *Hsp90* expression
Hsp90-qR	GAAGACACGGCGGACATA	
Hsp104-qF	AACCGAGCGTGCTGGAAA	Detects the *Hsp104* expression
Hsp104-qR	ATCAGGCAGACGCCGAGA	
Ugp-qF	AGATAACCTTGGTGCCG	Detects the *Ugp* expression
Ugp-qR	CGAATAGAGCCCTCGTAG	
Pgm-qF	GCCGTACAGATCATCCT	Detects the *Pgm* expression
Pgm-qR	GACTAGCGGTCAACAAA	
Pgi-qF	CGTAGCGGCGAGTGGAA	Detects the *Pgi* expression
Pgi-qR	ACGAAGTGGGCGGTCAA	
Tpp-qF	TGGGCATCAGCAAGAAT	Detects the *Tpp* expression
Tpp-qR	TGCGTTGACACGACCTT	
Tps-qF	GAAGATGACAGCGAAGC	Detects the *Tps* expression
Tps-qR	ACAGCACGCCAAGAAGA	
Cch-qF	TGTTTCGTTCGGGCTAC	Detects the *Cch* expression
Cch-qR	GCAGAAGATACGGCACTC	
Mid-qF	TGTCCGTAGCTTTATGCG	Detects the *Mid* expression
Mid-qR	GAAACCCTCCCGTCCAG	
Yvc-qF	GCGATTCATCAAGAGTT	Detects the *Yvc* expression
Yvc-qR	TTTGTGAGGGTCGTAGA	
Plc-qF	GAAGTTCGTGGTATGGC	Detects the *Plc* expression
Plc-qR	CAAATGCTGTGAACCCT	
Cam-qF	GGAGGCGTTCAAGGTGT	Detects the *Cam* expression
Cam-qR	CATCGCCATCAACATCA	
Cna1-qF	GAAGGAAGAGTTGGAGGAG	Detects the *Cna1* expression
Cna1-qR	CACGAAGCAAAGCGAAT	
Cna2-qF	GCCATAGACGATACAGG	Detects the *Cna2* expression
Cna2-qR	TTCGCCTCCTTAGATAG	

Crz-qF	AGCCAAGGCATACTCAA	Detects the *Crz* expression
Crz-qR	GGAAATAGGAATCCGTTA	
Camk1-qF	TTCGGAGGTTCAAGGAG	Detects the *Camk1* expression
Camk1-qR	GGAGGTCTTCGCCAACT	
Camk2-qF	GAACAGAGGGAGCAGGAG	Detects the *Camk2* expression
Camk2-qR	GGCAGCCAAGAGTGATG	
Camk3-qF	CGATAAGATGGGAGACG	Detects the *Camk3* expression
Camk3-qR	ACTGCGGATTCAGGTGT	

The sequences with lowercase letters ggatcc, gggccc, aagctt and agatct indicate the *BamH*I, *Apa*I, *Hind*III and *Bgl*II restriction sites, respectively.

Table S2 The GenBank accession numbers for genes used

Gene	GenBank accession numbers
Gpd promoter	KY924471
Tps	AWX94602
GAPDH	OP616031
Hsp60	OP616028
Hsp90	OP616029
Hsp104	OP616030
Ugp	OP616025
Pgm	OP616026
Pgi	OP616027
Tpp	OP616032
Cch	ON982458
Mid	ON982459
Yvc	ON982460
Plc	ON982461
Cam	OP021652
Cna1	OP021653
Cna2	OP021654
Crz	OP036679
Camk1	OP616022
Camk2	OP616023
Camk3	OP616024
